# Investigation of Linear and Nonlinear Properties of a Heartbeat Time Series Using Multiscale Rényi Entropy

**DOI:** 10.3390/e21080727

**Published:** 2019-07-25

**Authors:** Herbert F. Jelinek, David J. Cornforth, Mika P. Tarvainen, Kinda Khalaf

**Affiliations:** 1 Australian School of Advanced Medicine, Macquarie University, Sydney 2109, Australia; 2 School of Community Health, Charles Sturt University, Albury 2640, Australia; 3 School of Design, Communication and IT, University of Newcastle, Newcastle 2308, Australia; 4 Department of Applied Physics, University of Eastern Finland, 70210 Kuopio, Finland; 5 Department of Clinical Physiology and Nuclear Medicine, Kuopio University Hospital, 70210 Kuopio, Finland; 6 Department of Biomedical Engineering, Khalifa University, Abu Dhabi 127788, UAE

**Keywords:** heart rate variability, entropy, nonlinear dynamics, cardiac autonomic neuropathy, diabetes

## Abstract

The time series of interbeat intervals of the heart reveals much information about disease and disease progression. An area of intense research has been associated with cardiac autonomic neuropathy (CAN). In this work we have investigated the value of additional information derived from the magnitude, sign and acceleration of the *RR* intervals. When quantified using an entropy measure, these time series show statistically significant differences between disease classes of Normal, Early CAN and Definite CAN. In addition, pathophysiological characteristics of heartbeat dynamics provide information not only on the change in the system using the first difference but also the magnitude and direction of the change measured by the second difference (acceleration) with respect to sequence length. These additional measures provide disease categories to be discriminated and could prove useful for non-invasive diagnosis and understanding changes in heart rhythm associated with CAN.

## 1. Introduction

Biological signals, including electrocardiograms (ECG) or the electrical activity of the heart, exhibit complex dynamics which are characterized by nonlinearity and nonstationarity and often include random noise due to movement artefacts [1]. Heartbeat time series associated with health and disease have been extensively investigated, where time and frequency domains, as well as nonlinear methods, are being proposed and summarized in a number of communications [2,3,4,5,6,7,8,9,10,11].

Physiological dynamics of the heartbeat time series change with healthy aging [12,13] and disease, but also during different activities such as sleeping [14,15] and exercise [16,17,18,19,20,21]. Other changes in dynamics can be attributed to pathology including cardiovascular disease and heart failure [22,23,24], diabetes [25], depression [26,27] and Parkinson’s disease [24,28,29]. For all physiological and pathophysiological models of autonomic function, heart rate variability (HRV) is calculated from the cardiac interbeat intervals (IBI) of the time series. All models assume that the extrinsic modulation of the heartbeat by the autonomic nervous system (ANS) and the endocrine system affect HRV by either increasing the interbeat interval (parasympathetic influence), or decreasing the interbeat interval (sympathetic influence), or a combination of both. Disturbance of the ANS modulation by pathophysiological processes, such as oxidative stress, can then lead to the characteristic changes in sympathovagal input to the heart associated with cardiac autonomic neuropathy (CAN).

### 1.1. Heartbeat Interval Time Series

Non-invasive methods that are independent of patient cooperation are preferable in the diagnosis of CAN, but still require further research to confirm their sensitivity and specificity in stratification of CAN progression. The most common method currently used is heart rate variability analysis [30,31]. HRV is a useful indication of the health of the cardiovascular system, and is commonly used in assessing the regulation of cardiac autonomic function. HRV has been described by a variety of measures such as time domain, frequency domain, and non-linear dynamic (NLD) measures. However, time domain and power spectral density determination are not suitable for the analysis of non-linear and long-range correlated time signals [32]. Application of new signal processing techniques based on NLD, on the other hand, provides supplementary information (i.e., hidden underlying mechanisms) regarding physiological and pathophysiological processes involved in cardiovascular function and pathology. Two components of a time series in particular, being the sign and magnitude, allow further investigation into the characteristics of the time series and have been discussed previously [33,34,35]. 

### 1.2. Decomposition of the RR Interval Time Series

The current work is based on Ashkenazy [33], who introduced a decomposition algorithm of the *RR* interval time series by calculating the beat-to-beat increment or first difference (Δ*RR* = *RR*_n_ − *RR*_n−1_). The first difference series is then decomposed into the magnitude and sign of the increments (|Δ*RRRR*| and sign (signΔ*RR*) respectively). 

Here, we extend this work by introducing the acceleration, defined as the difference between two successive differences, i.e.,

(1) $$\Delta^{2}RR=\left(RR_{n}-RR_{n-1}\right)-\left(RR_{n-1}-RR_{n-2}\right)$$

(2) $$\Delta^{2}RR=RR_{n}-2RR_{n-1}+RR_{n-2}$$

Velocity is defined as the rate of change in the *RR* interval length and therefore the first order difference (∆*RR*). Acceleration is then the second order difference (∆^2^*RR*). The second difference or acceleration is a measure of the change of *RR* points with respect to time and indicates the instantaneous acceleration of the heart rate. We propose that acceleration represents an additional descriptive term for a time series. The scaling properties in sign, magnitude and acceleration can then be analyzed by HRV measures, which define the temporal organization of the original time series. Previously detrended fluctuation analysis (DFA) was applied to the sign and magnitude time series [33]. Here, we analyze, sign, magnitude and acceleration using the multiscale Rényi entropy [36,37,38]. 

### 1.3. The Rényi Entropy

Entropy measures can be used to quantify the diversity, uncertainty, or randomness of a system, and are hence considered as beneficial tools for analyzing nonlinear time series, including those of short duration, towards identifying underlying pathology [39,40,41]. Global entropy measures, such as approximate entropy (ApEn) [42] and sample entropy (SampEn) [43], were adapted from the correlation dimension [44,45] and Kolmogorov entropy [46]. *RR* time series, however, are multifactorial and display multiscale characteristics, and thus neither ApEn nor SampEn are ideal for such types of biosignal processing. Nonlinear, multiscale dynamic systems can however be described by scaling exponents [47], as well as several multiscale measures [48,49]. Rényi entropy has several advantages. The major advantage of Rényi entropy is that it is robust for short time series, nonlinearity and nonstationarity. The Rényi entropy introduced here also has the advantage of addressing how the probabilities are calculated by applying a density method rather than a histogram method, which is the standard for calculation of multiscale entropy [49].

In the current work we use the Rényi entropy, which generalizes the Shannon entropy [50] and is defined as:

(3) $$H\left(\alpha \right)=\frac{1}{1-\alpha }log_{2}\left(\sum_{i=1}^{n}p_{i}^{\alpha }\right)$$

where *p_i_* is the probability that a random variable takes a given value out of *n* values*,* and *α* is the order of the entropy measure [50]. *H(0)* is simply the logarithm of *n*. As *α* increases, the measures become more sensitive to the values occurring at higher probability and less to those occurring at lower probability, which provides a picture of the *RR* length distribution within a signal. The probability, *p*, can be estimated for any sub-sample of *RR* intervals, by considering the sub-sample as a point embedded in a multi-dimensional space. The sub-sample is assigned a density measure by evaluating other sub-samples in its vicinity. This addresses the coarse-graining problem for the determination of scaling behavior in biosignal time series inherent in previous applications of the entropy measures by applying a Gaussian kernel [49,51]. The Gaussian kernel is calculated as the sum of all contributions from other *RR* sub-samples with index *j*:

(4) $$\rho_{i}=\frac{1}{\sigma \sqrt{2\pi }}\sum_{j=1}^{n}e^{-\frac{dist_{ij}^{2}}{2\sigma^{2}}}$$

where *σ* is the dispersion of the function, and replaces the tolerance as suggested by Costa [48]. We designate the number of *RR* intervals in the sub-sample as π (not to be confused with the irrational number pi), and use the Euclidean distance measure in *π* dimensions:

(5) $$dist_{ij}=\sum_{k=0}^{\pi }{\left(x_{i+k}-x_{j+k}\right)}^{2}.$$

Here, we investigate the efficacy of applying multiscale Rényi entropy as a measure of HRV with respect to the sign, magnitude and the rate of change (acceleration) of the biosignal over time. 

## 2. Methods

### 2.1. Patient Selection

Heart rate tachograms were obtained from data collected at the Charles Sturt Diabetes Complications Screening Clinic (DiScRi), Australia [52] and were approved by the Charles Sturt University Human Ethics Committee. Written informed consent was obtained from all participants. A 20-min lead II ECG recording was taken from participants attending the clinic, using Powerlab hardware with Chart 7 software (ADInstruments, Sydney) during the morning in an ambient temperature room and after the participants were relaxed. Participants were comparable for age, gender, and heart rate, and after initial screening, those with heart disease, presence of a pacemaker, kidney disease or polypharmacy (including multiple anti-arrhythmic medications) were excluded from the study. The status of CAN was defined using the Cardiac Autonomic Reflex Test battery criteria [53]. Each participant was assigned as either without CAN (71 participants), early CAN (67 participants) or definite CAN (NN participants) [54,55]. 

### 2.2. ECG Recording and Obtaining the RR Intervals

**Figure 1:**
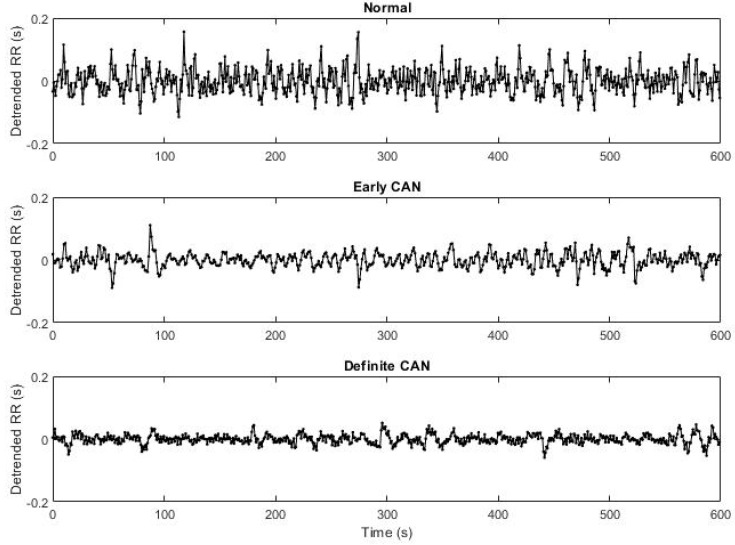
*RR* interval time series of normal, early cardiac autonomy neuropathy (eCAN) and definite CAN (dCAN).

From the 20-min *RR* tachogram, a 10 min segment was selected from the middle in order to remove transient start up artefacts and movement at the end of the recording. The *RR* intervals were then extracted from this shorter recording, and data were visually verified to not include any missed, extra or misaligned (including ectopic) beat detections. No other information was used in this study. The raw *RR* interval series for each participant was detrended based on smoothness priors formulation [56]. For the purposes of an initial examination of the *RR* interval recordings, we have selected one recording from each of these three classes as follows. For each participant class (Normal, Early and Definite), the Standard deviation of *RR* intervals in the time series were calculated. For each participant, we calculated the difference between this and the Median values of the Standard deviation obtained for all participants of the same class. We selected the *RR* time series closest to the median for that class. The 10 min *RR* time series for these representatives are shown in Figure 1. Horizontal scales are the same to allow comparison, but vertical scales are as indicated on each graph. Figure 1 shows that the participant from the Normal class manifested *RR* intervals with mostly low deviation from the mean, but some large excursions (standard deviation = 0.0357). In comparison, the participant from the Definite class showed fewer large excursions (*SD* = 0.0173), while the participant from the Early class was in between these (*SD* = 0.025926).

### 2.3. Decomposition

The *RR* interval time series was decomposed into increment, magnitude, sign and acceleration, as discussed above. Raw *RR* intervals were filtered and the trend was removed. Increments were calculated as the difference between successive *RR* intervals. The magnitude, sign and acceleration of the increments were then determined. Finally, the Rényi entropy was calculated for the sign, magnitude and acceleration time series, using a variety of values for parameter sequence length π, exponent α, and width of the kernel function σ. This results in four different measures:

- Rényi entropy calculated from a sequence of the magnitude of the difference in *RR* intervals
- Rényi entropy calculated from a sequence of the sign of the difference in *RR* intervals
- Rényi entropy calculated from a sequence of the acceleration of *RR* intervals

### 2.4. Calculating the Multiscale Rényi (MSRen) Entropy

The Rényi entropy was calculated for scaling exponents *α* of integer values from −5 to +5. The entropy values were then normalized by dividing by log_2_ of the number of length of the *RR* interval time series. A range of sequence lengths, π, was also used, and the dispersion of the Gaussian function (σ) was varied in proportion. Sequence lengths of 1, 2, 4, 8 and 16 *RR* intervals were adopted, with corresponding values of σ as 0.01, 0.02, 0.04, 0.08 and 0.16, respectively. A Mann-Whitney test was performed to compare the Rényi value obtained for the Normal to that obtained for the Early CAN group, and a similar comparison between the Early CAN and the Definite CAN group, and between the Definite CAN and the Normal group.

## 3. Results

**Figure 2:**
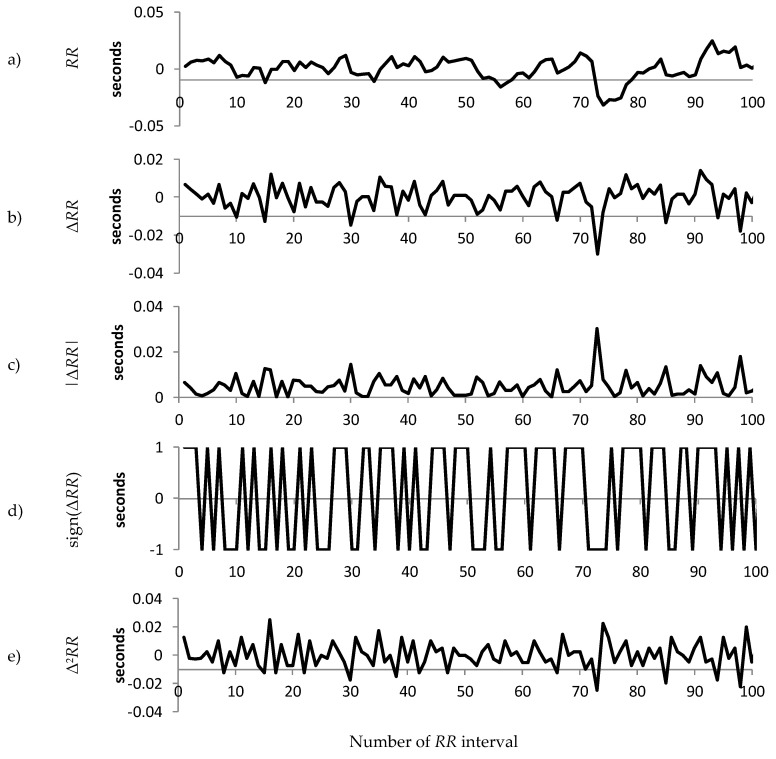
An illustration of the composition of the raw 100 *RR* tachogram for participants without CAN (Normal group). (**a**) The *RR* time series after filtering and pre-processing; (**b**) The increment (Δ*RR* = *RR*_n_ − *RR*_n−1_) of the time series shown in (a); (**c**) The magnitude of the increment; (**d**) The sign of the increment; (**e**) The acceleration (Δ^2^*RR* = *RR*_n_ − 2*RR*_n−1_ + *RR*_n−2_).

**Figure 3:**
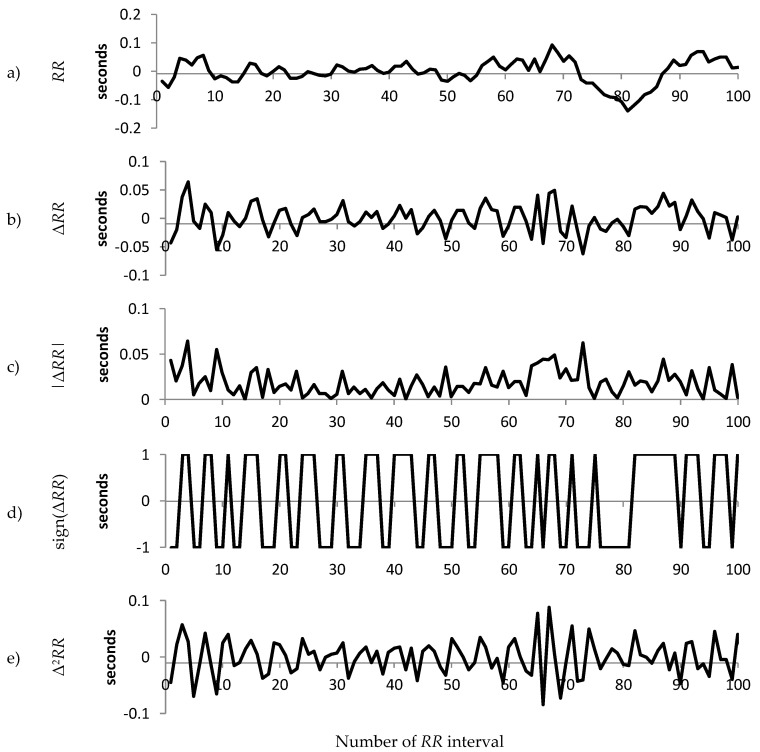
An illustration of the composition of the raw 100 *RR* tachogram for a participant with Early CAN. (**a**) The *RR* time series after filtering and pre-processing; (**b**) The increment (Δ*RR* = *RR*_n_ − *RR*_n−1_) of the time series shown in (a); (**c**) The magnitude of the increment; (**d**) The sign of the increment; (**e**) The acceleration (Δ^2^*RR* = *RR*_n_ − 2*RR*_n−1_ + *RR*_n−2_).

**Figure 4:**
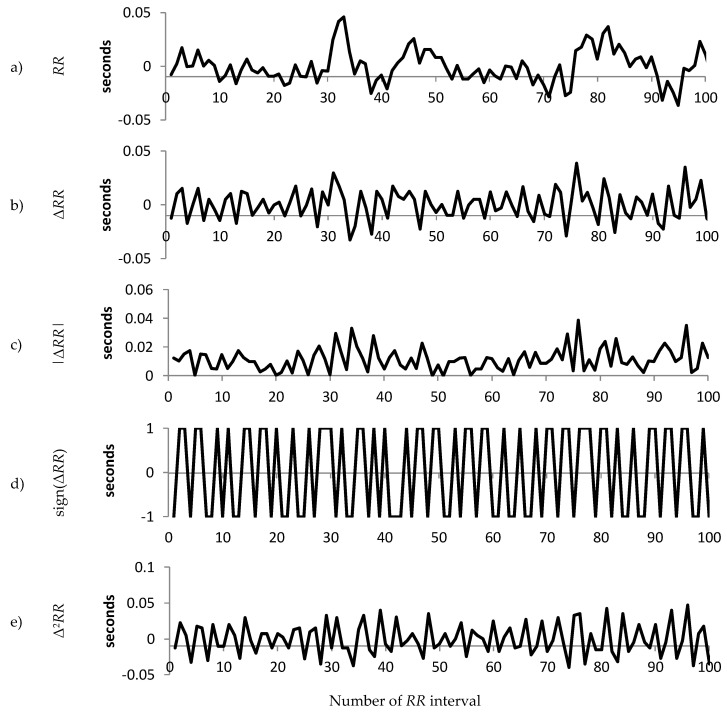
An illustration of the composition of the raw 100 *RR* tachogram for a person with Definite CAN. (**a**) The *RR* time series after filtering and pre-processing; (**b**) The increment (Δ*RR* = *RR*_n_ − *RR*_n−1_) of the time series shown in (a); (**c**) The magnitude of the increment; (**d**) The sign of the increment; (**e**) The acceleration (Δ^2^*RR* = *RR*_n_ − 2*RR*_n−1_ + *RR*_n−2_).

For each patient group, we calculated the median value of the standard deviation of the *RR* intervals and selected the patients with standard deviation of *RR* intervals closest to the median for the group. The resulting three representative patients are used to illustrate the differences in sign, magnitude and acceleration between Normal, Early CAN and Definite CAN. Figure 2, Figure 3 and Figure 4 present a sample of 100 filtered *RR* intervals and their decomposition, using data from the three representative groups to illustrate the effect of working with the sign of the *RR* interval, first and second difference. All vertical axes are numbered in seconds. It can be observed, for example, that the increment Δ*RR* (b) varies between ±0.2 s with excursions up to 0.5 s, while the acceleration (e) varies between ±0.7 ms. There are frequent reversals of sign (d) with some periods of a continuation of the same sign. 

Figure 3 shows similar information from the representative participant with early CAN. The range of variation in *RR* interval, Δ*RR* and |Δ*RR*| can be observed to be much smaller than those in Figure 2, indicating a smaller variance in the *RR* interval. In addition, the acceleration is large compared to the representative with early CAN in Figure 3. The sign (Figure 2e) shows fewer changes in direction compared to the representative with early CAN in Figure 3.

Figure 4 shows a sample of the information from a participant with definite CAN. The difference in *RR* intervals (Figure 4b,c) can be seen to be even smaller than the example shown in Figure 2 or Figure 3, while the acceleration (Figure 4e) also has a smaller range than the example from either the Normal or Early group. The sign is different to either of the previous examples, as there appears to be more frequent reversals in sign when compared to those examples, but there is less of a mixture of fast and slow changes in sign.

In order to quantify these differences, the variation of each time series was evaluated, for each participant in the study, using the Rényi entropy. A variety of values were used for the parameters (sequence length *π*, exponent *α* and width of the kernel function *σ*).

Our results comparing Normal (N), Early (E) and Definite (D) CAN, based on the magnitude of the difference of *RR* intervals for sequence length *π* set to 1, 2, 4, 8, 16, and for *α* = +5 applying the Mann-Whitney tests obtained the smallest *p*-value (*p* < 0.0001) for the Definite to Normal comparison with a sequence length of *π* = 1 values for *ΔRR*. Normal versus Early was best differentiated with longer sequences of length *π* = 4 or *π* = 8, whereas for Early versus Definite the optimal sequence length was again *π* = 1. Extracting the sign of a Δ*RR* sequence provides information on the linear aspects of the traces but the separation of the classes is less pronounced as seen in the figures above and results in the tables below and hence the *p*-values are much larger, indicating a lesser role of the linear characteristics of the signals in differentiating CAN progression. Only Normal to Early CAN was significantly different for a sequence length of *π* = 8, suggesting that the nonlinear, fractal-like characteristics may play a larger role in CAN development.

Separating the classes based on the acceleration of Δ*RR* increments results in the smallest *p*-value (8.13 × 10^−5^) obtained for the Mann-Whitney test comparing Definite CAN to Normal, and a sequence length π = 4. For acceleration, separation of CAN progression improves with sequence length up to *π* = 4, and then decreases again for all comparisons, except for Normal versus Early, where the best separation is seen using *π* = 16. However, the best overall comparative results were found with *π* = 4. 

Table 1 concerns the magnitude of differences (|Δ*RR*|) and shows *p*-values obtained from three Mann-Whitney tests comparing Normal to Early (NE), Early to Definite (ED) and Definite to Normal (DN), for different values of the Rényi parameters sequence length *π*, and exponent α. The width of the kernel function σ was always chosen as *π*/100. Nearly all of these *p*-values are significant at the *p* < 0.01 level, and some are extremely small, suggesting that these Rényi exponents show an effect for all three of these comparisons. 

In general, the smallest values are found for normal versus definite CAN as would be expected. However, the table suggests that some values of the Rényi parameters are better than others at demonstrating this effect. Generally, the sequence length of 2 provides the best separation for ED (early–definite) and DN (definite–normal), but using *π* = 4 provides the best separation for NE (normal–early). n.s.—not significant.

Table 2 illustrates results for acceleration and shows *p*-values obtained from three Mann-Whitney tests for different values of sequence length *π*, and exponent *α*. The figures show an optimum value for *π* = 4 and for a variety of values for *α*. For short sequence lengths, the *p*-value increases with increasing *α*. For longer sequences the opposite is true.

**Figure 5:**
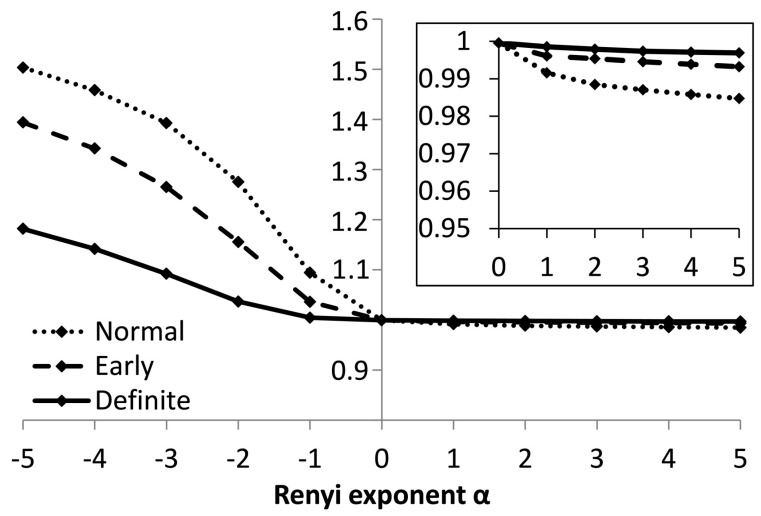
Values of Rényi entropy based on the magnitude of the difference between *RR* intervals, for sequences of 4 values.

The actual values of the Rényi entropy calculated from the magnitude of the increment of *RR* intervals |Δ*RR*|, using the parameters sequence length *π* = 4, and width of the kernel function σ = 0.04, are illustrated in Figure 5. The exponent *α* was varied so that −5 ≤ *α* ≤ 5. The inset highlights details of the exponents corresponding to the positive values of *α*. Rényi entropy calculated for the class of Early CAN lies in between those for the Normal and Definite classes.

**Figure 6:**
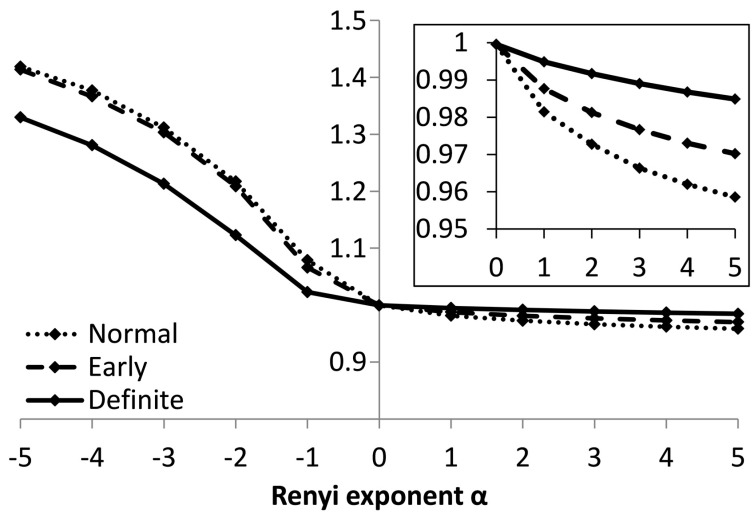
Values of Rényi entropy based on the acceleration of *RR* intervals, for sequences of 4 values.

Rényi entropy calculated from the acceleration of the *RR* intervals Δ^2^*RR*, using the parameters sequence length *π* = 4, and width of the kernel function σ = 0.04 indicates a better separation for positive *α* (Figure 6.). 

## 4. Discussion and Conclusions

In physiological dynamic systems, various extended concepts of entropy, such as approximate entropy (ApEn), sample entropy (SampEn), and multi-scale entropy have been developed to quantify various physiological signals [42,43,57]. The major advantage of Rényi entropy is that it is robust for short time series, nonlinearity and nonstationarity. The Rényi entropy introduced here also has the advantage of addressing how the probabilities are calculated by applying a density method based on a Gaussian kernel rather than a histogram method, which is the standard for calculation of multiscale entropy. *H(α)* is the order of the Rényi entropy measure. As *α* increases, the measures become more sensitive to the values occurring at higher probability and less to those occurring at lower probability, which provides a picture of the *RR* length distribution within a signal [49]. Including acceleration in our model then adds information about the heart rate variability by providing information not only on the change in the system using the first difference but also the magnitude and direction of the change measured by the second difference (acceleration) with respect to sequence length.

Heart rhythm is characterized by a scale-invariant, nonlinear dynamics displaying long-range power-law correlations over a range of time scales [58,59] akin to 1/f [60] or fractal-like scaling [1,39,47,57,58,61,62,63,64,65]. Fractal-like scaling analysis has been shown to indicate risk of adverse cardiac events [66,67]. 

Bio signals quantifying cardiac interbeat intervals (*RR* intervals) exhibit complex dynamics that vary with age and disease and can be characterized by scaling laws [12,68]. In healthy subjects, *RR* interval time signals present large variability, which is a function of the numerous physiological processes that influence heart rhythm, including ANS and neuroendocrine factors. Nonstationarity and nonlinear dynamics characteristic of these signals are believed to be due to the complex interaction between the two branches of the ANS, endocrine factors and the intrinsic cardiac control mechanisms. 

Early identification of CAN is crucial for more effective clinical outcomes. Studies have shown that the one of the earliest signs for a CAN diagnosis is the reduction of HRV. Thus, understanding of the time series characteristics and selecting an appropriate method to analyze these signals and interpret the results is paramount. A consistent finding of ours is that the most difficult two classes to separate were Definite and Early CAN. This implies that patients in the early stages of CAN have similar HRV features to those in the definite group. This may be a reflection that the existing CART criteria are somewhat conservative in identifying CAN, or the two blood pressure tests included in the CART battery indicative of sympathetic dysfunction do not clearly identify disease progression from early to definite CAN, and that sympathetic dysfunction may already be a factor in early CAN [69].

The typical *RR* tachogram consists of linear and nonlinear portions, which overlap and lead to the characteristic heart rate variability. In this work, we show that different components of the *RR* tachogram are able to differentiate between the stages of CAN progression from normal and early CAN to definite CAN. These different components rely on the fact that control of heart rate entails changes in both a positive and negative directions. In particular, the magnitude and acceleration of the changes in *RR* increments separate all three groups. Both of these series carry information on the nonlinear properties of the interbeat interval time series and indicate that fractal-like or power law dynamics within the biosignals become more prominent with disease progression. This complex behavior is further illustrated by the larger and more often occurring deviations in acceleration.

Recent NLD methods continue to shed light on HRV changes under various physiological and pathological conditions, providing valuable potential prognostic and diagnostic information and complementing traditional time- and frequency-domain analyses. With the advent of multiple tools and algorithms, it is critical to identify which of these methods should be selected and under which conditions they should be applied. Our work aligns with previous work, confirming the efficacy of complex measures for representing and quantifying heart rate variability. The current research has focused on investigating the differences in successive *RR* intervals adopting interbeat acceleration as a novel feature, which provides additional information about the nonlinearity of heartbeat regulation and hence the identification of disease. The high degree of separation obtained between classes of disease points to its diagnostic and risk stratification potential in cardiac autonomic neuropathy, and provides a much less invasive test for this disease, with the advantages of faster diagnosis, better access to treatment and more effective clinical outcomes.

## Figures and Tables

**Table 1:** Classification results based on Rényi exponents applied to the magnitude of differences (|Δ*RR*|). Figures represent *p*-values for the results of Mann-Whitney tests for comparisons of Normal to Early (NE), Early to Definite (ED) and Definite to Normal (DN). Values shown in bold are the smallest *p*-value for each comparison (Table 1), while tests that were not significant are indicated by n.s.

	Test	*π* = 1	*π* = 2	*π* = 4	*π* = 8	*π* = 16
*α* = 1	NE	0.0001	<0.0001	**<0.0001**	0.0001	0.002
	ED	0.004	**0.006**	0.02	n.s.	n.s.
	DN	**<0.0001**	<0.0001	0.0002	0.002	0.03
*α* = 2	NE	0.0002	<0.0001	**<0.0001**	<0.0001	0.0007
	ED	0.002	**0.004**	0.01	0.05	n.s.
	DN	<0.0001	**<0.0001**	0.0001	0.001	0.02
*α* = 3	NE	0.0002	<0.0001	**<0.0001**	<0.0001	0.0004
	ED	0.003	**0.005**	0.009	0.004	0.2
	DN	<0.0001	**<0.0001**	<0.0001	0.001	0.01
*α* = 4	NE	0.0003	<0.0001	**<0.0001**	<0.0001	0.0003
	ED	0.002	**0.005**	0.009	n.s.	n.s.
	DN	<0.0001	**<0.0001**	<0.0001	0.0009	0.007
*α* = 5	NE	0.0003	<0.0001	**<0.0001**	**<0.0001**	0.0002
	ED	**0.002**	0.005	0.009	0.02	n.s.
	DN	**<0.0001**	<0.0001	<0.0001	0.0007	0.006

**Table 2:** Classification results based on Rényi exponents applied to acceleration (|Δ^2^*RR*|). Figures represent *p*-values for the results of Mann-Whitney tests for comparisons of Normal to Early (NE), Early to Definite (ED) and Definite to Normal (DN). Values shown in bold are the smallest *p*-value for each comparison (Table 1), while tests that were not significant are indicated by n.s.

	Test	*π* = 1	*π* = 2	*π* = 4	*π* = 8	*π* = 16
*α* = 1	NE	0.003	0.0005	**0.0002**	0.0003	0.0005
	ED	n.s.	0.03	**0.01**	0.02	n.s.
	DN	0.00147	0.0002	**<0.0001**	0.0005	0.004
*α* = 2	NE	0.007	0.0008	**0.0003**	0.0004	0.0004
	ED	n.s.	0.02	**0.009**	0.02	0.05
	DN	0.002	0.0003	**<0.0001**	0.0003	0.001
*α* = 3	NE	0.01	0.001	0.0003	0.0005	**0.0003**
	ED	n.s.	0.03	**0.007**	0.02	0.04
	DN	0.002	0.0004	**<0.0001**	0.0002	0.001
*α* = 4	NE	0.01	0.0009	0.0004	0.0005	**0.0003**
	ED	n.s.	0.03	**0.007**	0.02	0.03
	DN	0.003	0.0004	**<0.0001**	0.0002	0.0009
*α* = 5	NE	0.01	0.001	0.0004	0.0006	**0.0002**
	ED	n.s.	0.03	**0.007**	0.02	0.03
	DN	0.0038	0.0004	**<0.0001**	0.0001	0.0006

## References

[1] Goldberger A.L., Amaral L.A.N., Hausdorff J.M., Ivanov P.C., Peng C.-K., Stanley H.E. (2002). Fractal dynamics in physiology: Alterations with disease and aging. Proc. Natl. Acad. Sci. USA.

[2] Costa M., Goldberger A.L., Peng C.-K. (2005). Multiscale entropy analysis of biological signals. Phys. Rev. E.

[3] Peng C.K., Havlin S., Stanley H.E., Goldberger A.L. (1995). Quantification of scaling exponents and cross over phenomena in nonstationary heartbeat time series analysis. Chaos.

[4] Ivanov P.C., Rosenblum M.G., Peng C.K., Mietus J., Havlin S., Stanley H.E., Goldberger A.L. (1996). Scaling behaviour of heartbeat intervals obtained by wavelet-based time-series analysis. Nature.

[5] Agelink M.W., Malessa R., Baumann B., Majewski T., Akila F., Zeit T., Ziegler D. (2001). Standardized tests of heart rate variability: Normal ranges obtained from 309 healthy humans, and effects of age, gender and heart rate. Clin. Auton. Res..

[6] Bellavere F., Balzani I., De Masi G., Carraro M., Carenza P., Cobelli C., Thomaseth K. (1992). Power spectral analysis of heart-rate variations improves assessment of diabetic cardiac autonomic neuropathy. Diabetes.

[7] Yeragani V.K., Srinivasan K., Vempati S., Pohl R., Balon R. (1993). Fractal dimension of heart rate time series: An effective measure of autonomic function. J. Appl. Physiol..

[8] Malik M., Camm J. (1995). HRV Variability.

[9] Electrophysiology, Task Force of the European Society of Cardiology the North American Society of Pacing (1996). Special report: Heart rate variability standards of measurement, physiological interpretation, and clinical use. Circulation.

[10] Teich M.C., Lowen S.B., Jost B.M., Vibe-Rheymer K. (2001). Heart Rate Variability: Measures and Models.

[11] Khandoker A.H., Jelinek H.F., Moritani T., Palaniswami M. (2010). Association of cardiac autonomic neuropathy with alteration of sympatho-vagal balance through heart rate variability analysis. Med. Eng. Phys..

[12] Pikkujämsä S.M., Mäkikallio T.H., Sourander L.B., Räihä I.J., Puukka P., Skyttä J., Peng C.K., Goldberger A.L., Huikuri H.V. (1999). Cardiac interbeat interval dynamics from childhood to senescence: Comparison of conventional and new measures based on fractals and chaos theory. Circulation.

[13] Schmitt D.T., Ivanov P.C. (2007). Fractal scale-invariant and nonlinear properties of cardiac dynamics remain stable with advanced age: A new mechanistic picture of cardiac control in healthy elderly. Am. J. Physiol..

[14] Burr R.L. (2007). Interpretation of normalised specrtal heart rate variability in sleep research: A critical review. Sleep.

[15] Vanoli E., Adamson P.B., Ba L., Pinna G.D., Lazzara R., Orr W.C. (1995). Heart rate variability during specific sleep stages. A comparison of healthy subjects with patients after myocardial infarction. Circulation.

[16] Hautala A.J., Makikallio T.H., Kiviniemi A., Laukkanen R.T., Nissila S., Huikuri H.V., Tulppo M.P. (2003). Cardiovascular autonomic function correlates with the response to aerobic training in healthy sedentary subjects. Am. J. Physiol. Heart Circ. Physiol..

[17] Jelinek H.F., Huang Z.Q., Khandoker A.H., Chang D., Kiat H. (2013). Cardiac rehabilitation outcomes following a 6-week program of PCI and CABG Patients. Front. Physiol..

[18] Kiviniemi A.M., Tulppo M.P., Eskelinen J.J., Savolainen A.M., Kapanen J., Heinonen I.H., Huikuri H.V., Hannukainen J.C., Kalliokoski K.K. (2014). Cardiac autonomic fucntion and high-intensity interval training in middle-aged men. Med. Sci. Sports Exerc..

[19] La Rovere M., Mortara A., Sandrone G., Lombardi F. (1992). Autonomic nervous system adaptations to short-term exercise training. Chest.

[20] Soares-Miranda L., Sandercock G., Valente H., Vale S., Santos R., Mota J. (2009). Vigorous physical activity and vagal modulation in young adults. Eur. J. Cardiovasc. Prevent. Rehab..

[21] Tulppo M.P., Mäkikallio T.H., Seppänen T., Laukkanen R.T., Huikuri H.V. (1998). Vagal modulation of heart rate during exercise: Effects of age and physical fitness. Am. J. Physiol. Heart Circ. Physiol..

[22] McLachlan C.S., Ocsan R., Spence I., Hambly B., Matthews S., Wang L., Jelinek H.F. (2010). Increased total heart rate variability and enhanced cardiac vagal autonomic activity in healthy humans with sinus bradycardia. Baylor University Medical Center Proceedings.

[23] Mäkikallio T.H., Huikuri H.V., Hintze U., Videbæk J., Mitrani R.D., Castellanos A., Myerburg R.J., Møller M., DIAMOND Study Group (2001). Fractal analysis and time- and frequency-domain measures of heart rate variability as predictors of mortality in patients with heart failure. Am. J. Cardiol..

[24] Huikuri H.V., Valkama J.O., Airaksinen K.E., Seppänen T., Kessler K.M., Takkunen J.T., Myerburg R.J. (1993). Frequency domain measures of heart rate variability before the onset of nonsustained and sustained ventricular tachycardia in patients with coronary artery disease. Circulation.

[25] Khandoker A.H., Jelinek H.F., Palaniswami M. Heart rate variability and complexity in people with diabetes associated cardiac autonomic neuropathy. Proceedings of the 2008 30th Annual International Conference of the IEEE Engineering in Medicine and Biology Society.

[26] Kemp A.H., Quintana D.S., Felmingham K.L., Matthews S., Jelinek H.F. (2012). Heart rate variability in unmedicated depressed patients without comorbid cardiovascular disease. PLoS ONE.

[27] Carney R.M., Freedland K.E. (2009). Depression and heart rate variability in patients with coronary artery disease. Clev. Clin. J. Med..

[28] Barbieri R., Citi L., Valenza G., Guerrisi M., Orsolini S., Tessa C., Diciotti S., Toschi N. (2013). Increased instability of heartbeat dynamics in Parkinson’s disease. Computing in Cardiology.

[29] Kallio M., Suominen K., Bianchi A.M., Mäkikallio T., Haapaniemi T., Astafiev S., Sotaniemi K.A., Myllylä V.V., Tolonen U. (2002). Comparison of heart rate variability analysis methods in patients with Parkinson’s disease. Med. Biol. Eng. Comput..

[30] Vinik A.I., Erbas T., Casellini C.M. (2013). Diabetic cardiac autonomic neuropathy, inflammtion and cariovascular disease. J. Diabetes Investig..

[31] Charles M., Fleischer J., Witte D.R., Ejskjaer N., Borch-Johnsen K., Lauritzen T., Sandbaek A. (2013). Impact of early detection and treatment of diabetes on the 6-year prevalence of cardiac autonomic neuropathy in people with screen-detected diabetes: ADDITION-Denmark, a cluster-randomised study. Diabetiologia.

[32] Hurst H.E. (1951). Long-term storage capacity of reservoirs. Trans. Am. Soc. Civ. Eng..

[33] Ashkenazy Y., Ivanov P.C., Havlin S., Peng C.K., Goldberger A.L., Stanley H.E. (2001). Magnitude and sign correlations in heartbeat fluctuations. Phys. Rev. Lett..

[34] Ashkenazy Y., Ivanov P.C., Havlin S., Peng C.K., Yamamoto Y., Goldberger A.L., Stanley H.E. (2000). Decomposition of heartbeat time series: Scaling analysis of the sign sequence. Comput. Cardiol..

[35] Ashkenazy Y., Lewkowicz M., Levitan J., Moelgaard H., Thomsen P.E.B., Saermark K. (1998). Discrimination of the healthy and sick cardiac autonomic nervous system by a new wavelet analysis of heartbeat intervals. Fractals.

[36] Jelinek H.F., Tarvainen M.P., Cornforth D.J. (2012). Renyi entropy in the identification of cardiac autonomic neuropathy in diabetes. Comput. Cardiol..

[37] Kurths J., Voss A., Saparin P., Witt A., Kleiner H.J., Wessel N. (1995). Quantitative analysis of heart rate variability. Chaos.

[38] Lake D.E. (2006). Renyi entropy measures of heart rate Gaussianity. IEEE Trans. Biomed. Eng..

[39] Voss A., Schulz S., Schroeder R., Baumert M., Caminal P. (2009). Methods derived from nonlinear dynamics for analysing heart rate variability. Phil. Trans. Math. Phys. Eng. Sci..

[40] Wessel N., Schumann A., Schirdewan A., Voss A., Kurths J. (2000). Entropy measures in heart rate variability data. International Symposium on Medical Data Analysis.

[41] Wessel N., Voss A., Malberg H., Ziehmann C., Voss H.U., Schirdewan A., Meyerfeldt U., Kurths J. (2000). Nonlinear analysis of complex phenomena in cardiological data. Herzschr. Elektrophys..

[42] Pincus S. (1991). Approximate entropy as a measure of system complexity. Proc. Nat. Acad. Sci. USA.

[43] Richman J.S., Moorman J.R. (2000). Physiological time-series analysis using approximate entropy and sample entropy. Am. J. Physiol. Heart Circ. Physiol..

[44] Grassberger P. (1988). Finite sample corrections to entropy and dimension estimates. Phys. Lett. A.

[45] Grassberger P., Procaccia I. (1983). Measuring the strangeness of strange attractors. Physica.

[46] Eckmann J.P., Ruelle D. (1985). Ergodic theory of chaos and strange attractors. The Theory of Chaotic Attractors.

[47] Ivanov P.C., Amaral L.A.N., Goldberger A.L., Havlin S., Rosenblum M.G., Struzik Z.R., Stanley H.E. (1999). Multifractality in human heartbeat dynamics. Nature.

[48] Costa M., Goldberger A.L., Peng C.-K. (2002). Multiscale entropy analysis of complex physiological time series. Phys. Rev. Lett..

[49] Cornforth D., Tarvainen M., Jelinek H.F. (2014). How to Calculate Renyi Entropy from Heart Rate Variability, and Why it Matters for Detecting Cardiac Autonomic Neuropathy. Front. Bioeng. Biotechnol..

[50] Rényi A. (1961). On measures of information and entropy. Proceedings of the Fourth Berkeley Symposium on Mathematics, Statistics and Probability.

[51] Xu Y., Ma Q.D.Y., Schmitt D.T., Bernaola-Galván P., Ivanov P.C. (2011). Effects of coarse-graining on the scaling behavior of long-range correlated and anti-correlated signals. Phys. A Stat. Mech. Appl..

[52] Jelinek H.F., Wilding C., Tinley P. (2006). An innovative multi-disciplinary diabetes complications screening programme in a rural community: A description and preliminary results of the screening. Aust. J. Prim. Health.

[53] Spallone V., Bellavere F., Scionti L., Maule S., Quadri R., Bax G., Melga P., Viviani G.L., Esposito K., Morganti R. (2011). Recommendations for the use of cardiovascular tests in diagnosing diabetic autonomic neuropathy. Nutr. Metab. Cardiovasc. Dis..

[54] Pop-Busui R., Evans G.W., Gerstein H.C., Fonseca V., Fleg J.L., Hoogwerf B.J., Genuth S., Grimm R.H., Corson M.A., Prineas R. (2010). The ACCORD Study Group. Effects of cardiac autonomic dysfunction on mortality risk in the Action to Control Cardiovascular Risk in Diabetes (ACCORD) Trial. Diabetes Care.

[55] Flynn A.C., Jelinek H.F., Smith M.C. (2005). Heart rate variability analysis: A useful assessment tool for diabetes associated cardiac dysfunction in rural and remote areas. Aust. J. Rural Health.

[56] Tarvainen M.P., Ranta-Aho P.O., Karjalainen P.A. (2002). An advanced detrending method with application to HRV analysis. IEEE Trans. Biomed. Eng..

[57] Costa M., Goldberger A.L., Peng C.K. (2003). Multiscale entropy analysis: A new measure of complexity loss in heart failure. J. Electrocardiol..

[58] Gao J., Gurbaxani B.M., Hu J., Heilman K.J., Emauele V.A., Lewis G.F., Davila M., Unger E.R., Lin J.M.S. (2013). Multiscale analysis of heart rate variability in nonstationary environments. Front. Physiol..

[59] Saul J.P., Albrecht P., Berger R.D., Cohen R.J. (1988). Analysis of long term heart rate variability: Methods, 1/f scaling and implications. Pharmacology.

[60] Kobayashi M., Musha T. (1982). 1/f fluctuation of heart beat period. IEEE Trans. Biomed. Eng..

[61] Struzik Z.R., Hayano J., Sakata S., Kwak S., Yamamoto Y. (2004). 1/f Scaling in heartrate requires antagonistic autonomic control. Phys. Rev. E.

[62] Hu J., Gao J., Tung W.-W., Cao Y. (2010). Multiscale analysis of heart rate variability: A comparison of different complexity measures. Ann. Biomed. Eng..

[63] Kiyono A., Struzik Z.R., Aoyagi N., Yamamoto Y. (2006). Multiscale probability density function analysis: Non-Gaussian and scale-invariant fluctuations of healthy human HRV. IEEE Trans. Biomed. Eng..

[64] Thurner S., Feurstein M.C., Teich M.C. (1998). Multiresolution wavelet analysis of heartbeat intervals discriminates healthy patients from those with cardiac pathology. Phys. Rev. Lett..

[65] Krstacic G., Krstacic A., Smalcelj A., Milicic D., Jembrek-Gostovic M. (2007). The “Chaos Theory” and nonlinear dynamics in heart rate variability analysis: Does it work in short-time series in patients with coronary heart disease?. Ann. Noninvasive Electrocardiol..

[66] Ho K.K., Moody G.B., Peng C.K., Mietus J.E., Larson M.G., Levy D., Goldberger A.L. (1997). Predicting survival in heart failure case and control subjects by use of fully automated methods for deriving nonlinear and conventional indices of heart rate dynamics. Circulation.

[67] Laitio T., Jalonen J., Kuusela T., Scheinin H. (2007). The role of heart rate variability in risk stratification for adverse postoperative cardiac events. Anesth. Analg..

[68] Goldberger A.L. (1996). Non-linear dynamics for clinicians: Chaos theory, fractals, and complexity at the bedside. Lancet.

[69] Bellavere F., Bosello G., Fedele D., Cardone C., Ferri M. (1983). Diagnosis and management of diabetic autonomic neuropathy. BMJ.

